# Cerebral magnetic resonance imaging of coincidental infarction and small vessel disease in retinal artery occlusion

**DOI:** 10.1038/s41598-020-80014-9

**Published:** 2021-01-13

**Authors:** Yong Dae Kim, Jun Yup Kim, Young Joo Park, Sang Jun Park, Sung Hyun Baik, Jihoon Kang, Cheolkyu Jung, Se Joon Woo

**Affiliations:** 1 Department of Ophthalmology, Seoul National University College of Medicine, Seoul National University Bundang Hospital, 173-82 Gumi-ro, Bundang-gu, Seongnam-si, Gyeonggi-do 13620 South Korea; 2 Department of Ophthalmology, Kangdong Sacred Heart Hospital, Seoul, South Korea; 3 Department of Radiology, Seoul National University College of Medicine, Seoul National University Bundang Hospital, 173-82 Gumi-ro, Bundang-gu, Seongnam-si, Gyeonggi-do 13620 South Korea; 4 Department of Neurology, Seoul National University College of Medicine, Seoul National University Bundang Hospital, Seongnam, South Korea; 5 Department of Ophthalmology, Kangwon National University Hospital, Chuncheon, South Korea

**Keywords:** Brain imaging, Retinal diseases

## Abstract

There are several reports in the literature on the association between non-arteritic retinal artery occlusion (NA-RAO) and acute ischemic stroke. We investigated the burden of small vessel disease (SVD) and cerebral coincident infarction observed on cerebral magnetic resonance imaging (MRI) in patients with newly diagnosed NA-RAO. In this retrospective, observational, case-series study, consecutive patients with NA-RAO who underwent cerebral MRI within one month of diagnosis between September 2003 and October 2018 were included. The classification of NA-RAO was based on ophthalmologic and systemic examinations. We also investigated the co-incident infarction and burden of underlying SVD, which were categorized as white matter hyperintensity lesion (WMH), cerebral microbleeds (CMB), and silent lacunar infarction (SLI). Among the 272 patients enrolled in the study, 18% presented co-incident infarction and 73% had SVD, which included WMH (70%), CMB (14%), and SLI (30%). Co-incident infarction, WMH, and SLI significantly increased with age: co-incident infarction was observed in 8% of young (< 50 years) patients and 30% of old (≥ 70 years) patients. The embolic etiology of RAO (large artery atherosclerosis, cardioembolism, and undetermined etiology) was significantly associated with the prevalence of SVD (82%: 70%: 64%, *P* = 0.002) and co-incident infarction (30%: 19%: 8%; *P* = 0.009). Therefore, high co-incidence of acute cerebral infarction and underlying SVD burden warrant careful neurologic examination and appropriate brain imaging, followed by management of NA-RAO. Urgent brain imaging is particularly pertinent in elderly patients with NA-RAO.

## Introduction

Retinal artery occlusion (RAO) results in sudden and catastrophic, total or segmental loss of vision^1^. Iatrogenic accident, trauma, or vasculitis may cause RAO; however, the most common etiology of non-arteritic RAO (NA-RAO)  is thromboembolism from a large artery or the heart^1–5^. Since the retina and brain have a common blood supply, namely the internal carotid artery (ICA), the thromboembolic event leads to ischemic stroke in the brain and NA-RAO in the retina^6,7^. Since NA-RAO has a similar pathogenesis and incidence pattern as ischemic stroke, it is regarded as one of the manifestations of ischemic stroke^2,6,7^. Therefore, cerebrovascular risk assessment and appropriate preventive management should be provided for subsequent ischemic stroke in emergency health care systems^2,8–10^. Furthermore, studies have reported that NA-RAO increased the risk for subsequent stroke in prior studies; thus, careful examinations of the source of embolization are essential to prevent cerebral vascular events^2,9,11^.

 There is no established guideline regarding systemic evaluation in patients with NA-RAO and application of cerebral MRI^12,13^. For proper management of NA-RAO, laboratory evaluations including hemoglobin A1c fraction, lipid profiles, erythrocyte sedimentation rate, C-reactive protein level, and complete blood count should be carefully checked^12,13^. Also, embolic source evaluations with carotid doppler or angiography with computed tomography/MR, electrocardiogram and echocardiography are required^12,13^. Because NA-RAO and ischemic stroke had similar pathogenesis, asymptomatic or symptomatic acute ischemic stroke lesions were usually combined in the NA-RAO. Therefore, urgent MRI with diffusion weighted imaging (DWI) for the evaluation of acute ischemic lesions and MR angiography (MRA) should be performed first for the purpose of secondary prevention of ischemic stroke in NA-RAO^6^. However, in addition to diffusion MRI, it is unclear whether MR sequences that can find other old silent brain lesions are also urgently needed for patients with NA-RAO.

Several studies have reported MRI findings including acute ischemic and old silent lesions in patients with NA-RAO^10,12–17^. Limitations of previous studies include the following: relatively small study population, limited to a specific location, or limited to branch RAO (BRAO)^13–16,18^. Moreover, most RAO studies have primarily focused on only acute cerebral ischemic lesions^14,16,17^. Other silent lesions, also known as cerebral small vessel diseases (SVD), can be frequently observed on cerebral MRI. SVDs are prevalent, particularly in the elderly population, and collectively refer to white matter hyperintensities (WMH), silent lacunar infarctions (SLI), and cerebral microbleeds (CMB)^19^. WMH and SLI are the most acknowledged cerebral MRI features in SVD^20,21^. CMB is also considered to be one of the consequences of SVDs, particularly those caused by vessel rupture, which results in perivascular collection of hemosiderin deposits, representing the foci of previous hemorrhage^22,23^. SVDs are pathologic events that affect cognitive function and are strongly linked with a future risk of stroke^18,24–27^. However, the importance of the prevalence of SVD in patients with NA-RAO, which shares common pathogenesis with ischemic stroke, have been underestimated^15^.

There was a few small studies on the relationship between retinal vascular changes and cerebral SVD^28–30^, and it has been suggested that retinal vein occlusion and non-arteritic ischemic optic neuropathy are related with cerebral SVD^31,32^. However, it is unknown how many SVDs are found in patients with NA-RAO, and in which type of etiology of NA-RAO is commonly related to which type of SVDs. The reason for the lack of research on the link between NA-RAO and SVD is that SVD is an asymptomatic lesion, and SVD is not directly related with the early risk of ischemic stroke after occurrence of RAO. Therefore, larger studies are needed to elucidate the role of concurrent brain lesions occurring at the time of RAO diagnosis, which may provide a deeper understanding of the cerebral vascular diseases associated with RAO and the need for systemic evaluation, including cerebral MRI.

We aimed to investigate the concurrent brain lesions in patients with NA-RAO who underwent cerebral MRI within one month of diagnosis and analyzed the associated clinical factors. Additionally, subgroup analysis was performed to determine whether there were differences in brain lesions based on RAO types and etiologyies. We also analyzed the clinical significance of cerebral MRI in patients with NA-RAO.

## Methods

### Subjects

Consecutive patients with NA-RAO who underwent cerebral MRI to evaluate the concurrent neurological abnormalities within a month of diagnosis at Seoul National University Bundang Hospital (SNUBH) between September 2003 and October 2018 were included in this study. Cerebral MRI was immediately and routinely recommended for all patients at the time of diagnosis, and it was performed in patients who had been informed and agreed to undergo the exam. Patients with the presence of any of the following criteria were excluded from the study: (1) arteritic RAO, (2) iatrogenic or traumatic RAOs, including those associated with accidental intravascular facial filler injection or due to orbital trauma, and (3) combined retinal vein occlusion.

### Demographics, ophthalmic evaluation, and systemic evaluation

All patients underwent slit-lamp biomicroscopy, indirect fundus examination, fundus photography (Vx-10; Kowa Optimed, Tokyo, Japan, or Optos PLC, Dunfermline, Scotland, UK), and fluorescein angiography (FA) (Vx-10 or Optos PLC) at the initial visit. We then categorized the included patients into two groups based on the initial ophthalmic examination: a BRAO group and a central RAO (CRAO) group. The CRAO group was further subcategorized into incomplete CRAO (diminished visual acuity with an unclear cherry-red spot and slight retinal edema), and complete CRAO (subtotal or total CRAO, severe reduction of visual acuity with marked cherry-red spot and distinct retinal edema) groups^33^. Two experienced ophthalmologists (YDK and YJP), who were blinded to the subjects’ identity and MRI results, evaluated the fundus photography and FA to classify RAO. Any disagreements were resolved via discussion and, if necessary, by consulting an additional grader (SJW). We also collected patient demographics and medical histories (e.g., medical history of hypertension, diabetes mellitus, dyslipidemia, smoking, and previous cardiovascular disease, including acute stroke, transient ischemic attack, valvular heart disease or equivalent condition, atrial fibrillation, and coronary artery disease) to assess cardio-/cerebrovascular risk factors. Electrocardiography, echocardiography, magnetic resonance angiography (MRA), coronary computed tomographic angiography, and other laboratory examinations were performed to identify the embolic source. MRA was performed in all patients concurrently with MRI. We measured the degree of carotid artery stenosis according to the North American Symptomatic Carotid Endarterectomy Trial (NASCET) criteria and defined symptomatic significant stenosis as a possible cause of arterial embolism if the degree of stenosis was 50% or higher^34^. The mechanism of RAO was classified into large artery atherosclerosis (LAA), cardioembolism, and undetermined etiology based on presumptive Trial of ORG 10,172 in Acute Stroke Treatment (TOAST) criteria^35^. LAA was adjudicated when RAO was assumed to have resulted from arterial embolism via the ipsilateral ICA atherosclerosis, cardioembolism by embolism from the cardiac disease, including atrial fibrillation, heart failure, and left ventricular wall motion abnormality, and undetermined etiology by embolism from two or more sources, or a negative source in the standard work-up.

### Cerebral magnetic resonance imaging

We assessed the cerebrovascular status by grading the initial cerebral MRI data using a 1.5 T or 3.0 T system (Intera or Achieva; Philips Medical Systems, Best, The Netherlands; or Signa Horizon). The whole brain was scanned with a slice thickness of 5.0 mm in the axial plane using T1-weighted images [repetition time (TR)/echo time (TE) = 300/10], T2-weighted images (TR/TE = 4800/100), fluid-attenuated inversion recovery (FLAIR) images (TR/TE = 11,000/140), T2 fast field echo images (TR/TE = 724/23), three-dimensional time of flight MRA images (TR/TE = 20/7, slice thickness = 1.2 mm), and gadolinium-enhanced MRA (TR/TE = 3.84–5.7/1.49–2.0, slice thickness = 0.5 mm, flow rate = 2 mL/s, dose = 0.1 mmol/kg). DWI (TR/TE = 4800/66) was obtained in most patients (n = 244/272, 89%) to evaluate an acute infarct, which was defined as focal diffusion-restricted lesions on DWI^36^.

**Figure 1:**
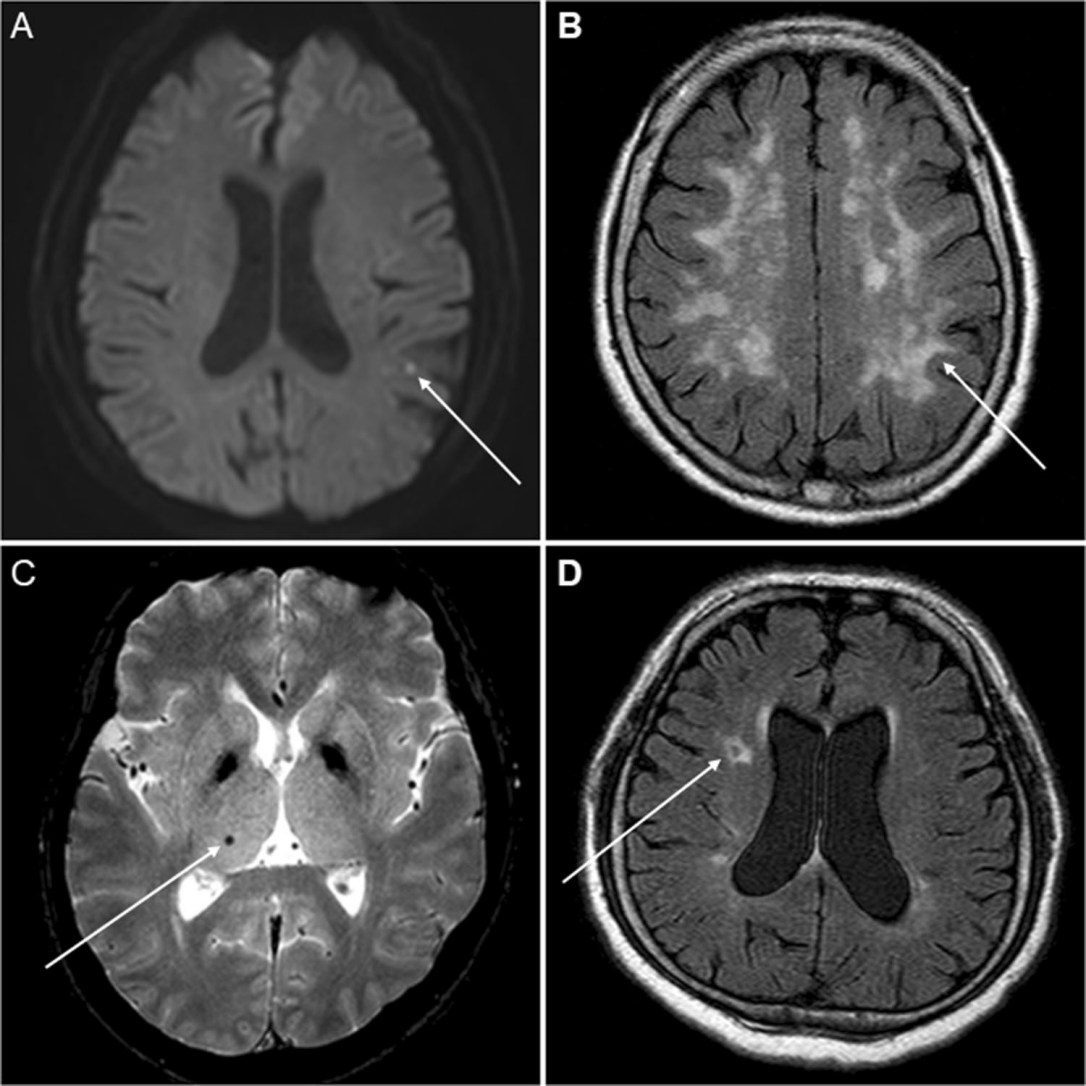
Representative cerebral magnetic resonance imaging of (**A**). acute infarction, (**B**). white matter hyperintensity lesion, (**C**). cerebral microbleeds, and (**D**). silent lacunar infarction. Arrows indicate the corresponding lesion in each figure.

Cerebral SVD was divided into WMH, CMB, and SLI. WMH were defined as areas of bright, high-signal intensities noted on T2-weighted images, and were classified into four severity groups based on their Fazekas score: grade 0 (absent), grade 1 (caps or pencil-thin lining), grade 2 (smooth halo), and grade 3 (irregular periventricular signal extending into the deep white matter) ^37^. We defined CMB as black, round lesions with a blooming effect on gradient-recalled echo MRI, devoid of T1-weighted or T2-weighted hyper-intensity, with a minimum of half of the lesion surrounded by brain parenchyma between 1 and 5 mm in diameter^38^. SLI was defined as a focal lesion in the deep perforator territory, which was ≥ 3 mm and ≤ 15 mm in diameter, with a high signal on T2-weighted images or FLAIR images and a low signal on T1-weighted images, often surrounded by a high signal rim on FLAIR images. Based on this classification, we defined cerebral SVD as one of the following: grade 1 or greater WMH, presence of CMB, or presence of SLI. Figure 1 shows representative images of MRI findings, including acute infarct, WMH, CMB, and SLI. One experienced neuroradiologist who was blinded to all subjects' ophthalmologic diagnosis or examinations reviewed the MR images. A trained stroke neurologist (JYK) assessed the degree of WMH, CMB, and SLI on the cerebral MRI and carotid stenosis status on the MRA images.

### Statistical analysis

We used SPSS version 25.0 for Windows (SPSS, Inc., Chicago, IL, USA) for statistical analyses. A *P* value < 0.05 was considered statistically significant. An analysis was performed using Pearson's χ^2^-test or trend analysis (linear-by-linear association) for categorical variables, and an independent *t*-test or analysis of variance (ANOVA) for continuous variables as appropriate. We divided our patients into subgroups based on the type of RAO (BRAO, complete or incomplete CRAO), the etiology of RAO (LAA, cardioembolism, or undetermined etiology), and age by decade (< 50 years; 50–60 years; 60–70 years; ≥ 70 years). The demographics, comorbidities, and cerebral MRI findings were compared between CRAO and BRAO, etiology of RAO, and age groups, respectively. Multiple logistic regression analysis (backward elimination) was conducted to identify independent predictors of the co-incident cerebral infarction, WMH, and SLI on cerebral MRI. Conventional risk factors including age, sex, hypertension, diabetes, dyslipidemia, and obesity were considered in the uni- and multivariable analysis.

### Ethics statement

This retrospective study was approved by the Institutional Review Board (IRB) of the SNUBH (IRB B-1811/504–101), and the requirement of informed consent was waived from the IRB. The study complied with the guidelines of the Declaration of Helsinki.

## Results

### Demographics and clinical characteristics

A total of 272 patients with RAO were enrolled and categorized into the CRAO group (n = 190) and BRAO (n = 82) groups. Demographics, clinical characteristics, and embolic etiology are presented in Table 1. The mean age of the participants was 64.7 ± 14.5 years (range, 20–93 years), and the time from the onset of symptoms to initial cerebral MRI examination was 4.3 ± 7.5 days. A total of 226 patients (83%) underwent MRI within 1 week of symptom onset, and 244 patients (90%) underwent MRI within 2 weeks. The mean time from onset of symptoms to MRI was significantly less in the CRAO group (3.2 ± 6.0 days) than in the BRAO group (6.9 ± 9.8 days) (*P* < 0.001). Hypertension was the most common underlying condition (n = 163, 60%), followed by dyslipidemia (n = 79, 29%), obesity (n = 71, 26%), and diabetes mellitus (n = 54, 20%). There was no significant difference between the CRAO and BRAO groups in the prevalence of hypertension, diabetes mellitus, dyslipidemia, and smoking status. However, patients with CRAO were more likely to have had previous coronary artery diseases (*P* = 0.008), or valvular heart disease and/or atrial fibrillation (*P* = 0.015) than those with BRAO.

**Table 1:** Baseline clinical characteristics of patients with central retinal artery occlusion (CRAO) and patients with branch retinal artery occlusion (BRAO).

Variables	Total	CRAO versus BRAO		
	RAO (n = 272)	CRAO (n = 190)	BRAO (n = 82)	*P* value
Age (year), mean ± SD	64.7 ± 14.5	65.0 ± 14.0	64.0 ± 15.5	0.628
Male sex, n (%)	169 (62%)	114 (60%)	55 (67%)	0.270
Time from symptom onset to initial brain imaging (day) , mean ± SD	4.3 ± 7.5	3.1 ± 6.0	6.9 ± 9.8	** < 0.001**
Comorbidity				
Hypertension, n (%)	163 (60%)	109 (57%)	54 (66%)	0.190
Diabetes mellitus, n (%)	54 (20%)	41 (22%)	13 (16%)	0.277
Dyslipidemia, n (%)	79 (29%)	56 (29%)	23 (28%)	0.812
Obesity, n (%)	71 (26%)	46 (24%)	25 (30%)	0.279
Smoking, n (%)				
Current smoker	43 (16%)	32 (17%)	11 (13%)	0.431^a^
Ex-smoker	45 (17%)	32 (17%)	13 (16%)	
Never	184 (68%)	126 (66%)	58 (71%)	
Coronary artery disease, n (%)	40 (15%)	35 (18%)	5 (6%)	**0.008**
Valvular heart disease or Atrial fibrillation, n (%)	46 (17%)	39 (21%)	7 (9%)	**0.015**
History of stroke or TIA, n (%)	27 (10%)	21 (11%)	6 (7%)	0.344
Etiologic subtypes				
Large artery atherosclerosis	126 (46%)	88 (46%)	38 (46%)	0.997
Cardioembolism	37 (14%)	32 (17%)	5 (6%)	**0.018**
Other determined	2 (1%)	2 (1%)	0 (0%)	0.351
Undetermined	107 (39%)	68 (36%)	39 (48%)	0.068
Two or more	4 (2%)	2 (1%)	2 (2%)	0.383
Negative	73 (27%)	48 (25%)	25 (30%)	0.372
Incomplete	30 (11%)	18 (9%)	12 (15%)	0.212

Data are presented as number (%) or mean ± standard deviation. *P* < 0.05 was deemed to indicate clinical significance, values in boldface are statistically significant.

TIA = transient ischemic attack.

^a^Linear-by-linear association.

Approximately half of patients with RAO had an etiology of LAA (46%), followed by undetermined etiology (39%), and cardioembolism (14%). The embolism that originated from the LAA was not significantly different between the CRAO and BRAO groups (88/190, 46% vs. 38/82, 46%; *P* = 0.997); however, cardioembolism was significantly higher in the CRAO group than in the BRAO group (32/190, 17% vs. 5/82, 6%; *P* = 0.018).

### Cerebral MRI findings

The comparison of the detailed cerebral MRI findings between CRAO and BRAO in patients with total RAO are summarized in Table 2. Among patients who underwent DWI (n = 244), co-incident acute cerebral infarction was observed in 49 (18%) subjects. The type of RAO was not significantly associated with co-incident cerebral infarction (19% of CRAO and 15% of BRAO, *P* = 0.467).

**Table 2:** Comparison of brain MRI findings between patients with central retinal artery occlusion (CRAO) and patients with branch retinal artery occlusion (BRAO).

	Total	CRAO versus BRAO		
	RAO (n = 272)	CRAO (n = 190)	BRAO (n = 82)	*P* value
Diffusion weighted image, n (%)	244 (89%)	174 (92%)	70 (85%)	
Co-incident cerebral infarction, n (%)	49 (18%)	37 (19%)	12 (15%)	0.467
Cerebral small vessel disease	198 (73%)	142 (75%)	56 (68%)	0.273
White matter hyperintensity, n (%)	188 (69%)	136 (72%)	52 (63%)	0.181
Grade 1	128 (47%)	96 (51%)	32 (39%)	0.614*
Grade 2	49 (18%)	32 (17%)	17 (21%)	
Grade 3	11 (4%)	8 (4%)	3 (4%)	
Cerebral microbleeds, n (%)	38 (14%)	28 (15%)	10 (12%)	0.582
Silent lacunar infarct, n (%)	82 (30%)	59 (31%)	23 (28%)	0.620
MRA—ICA involvement	138 (51%)	96 (51%)	42 (51%)	0.916
Mild stenosis	77 (28%)	52 (27%)	25 (30%)	0.838^a^
Moderate to severe stenosis	61 (22%)	44 (23%)	17 (21%)	

Data are presented as number (%). *P* < 0.05 was deemed to indicate clinical significance, values in boldface are statistically significant.

MRI = magnetic resonance imaging, MRA = magnetic resonance angiography, ICA = internal carotid artery.

^a^Linear-by-linear association.

The overall prevalence of cerebral SVD was 73% (n = 198/272) in total RAO patients, without any significant difference between the CRAO and BRAO groups (*P* = 0.273). The most common SVD type was WMH, specifically grade 1 WMH (n = 128/272; 47%), followed by grade 2 (n = 49/272; 18%), and grade 3 (n = 11/272; 4%) WMH. SLI and CMB were observed in 30% (n = 82/272) and 14% (n = 38/272) of the RAO patients, respectively. There were no significant differences between the CRAO and BRAO groups for each category of SVD. The comparison between complete CRAO group and incomplete CRAO group was also performed; however, clinical characteristics and MRI findings showed no significant differences (Supplementary Table S1).

**Figure 2:**
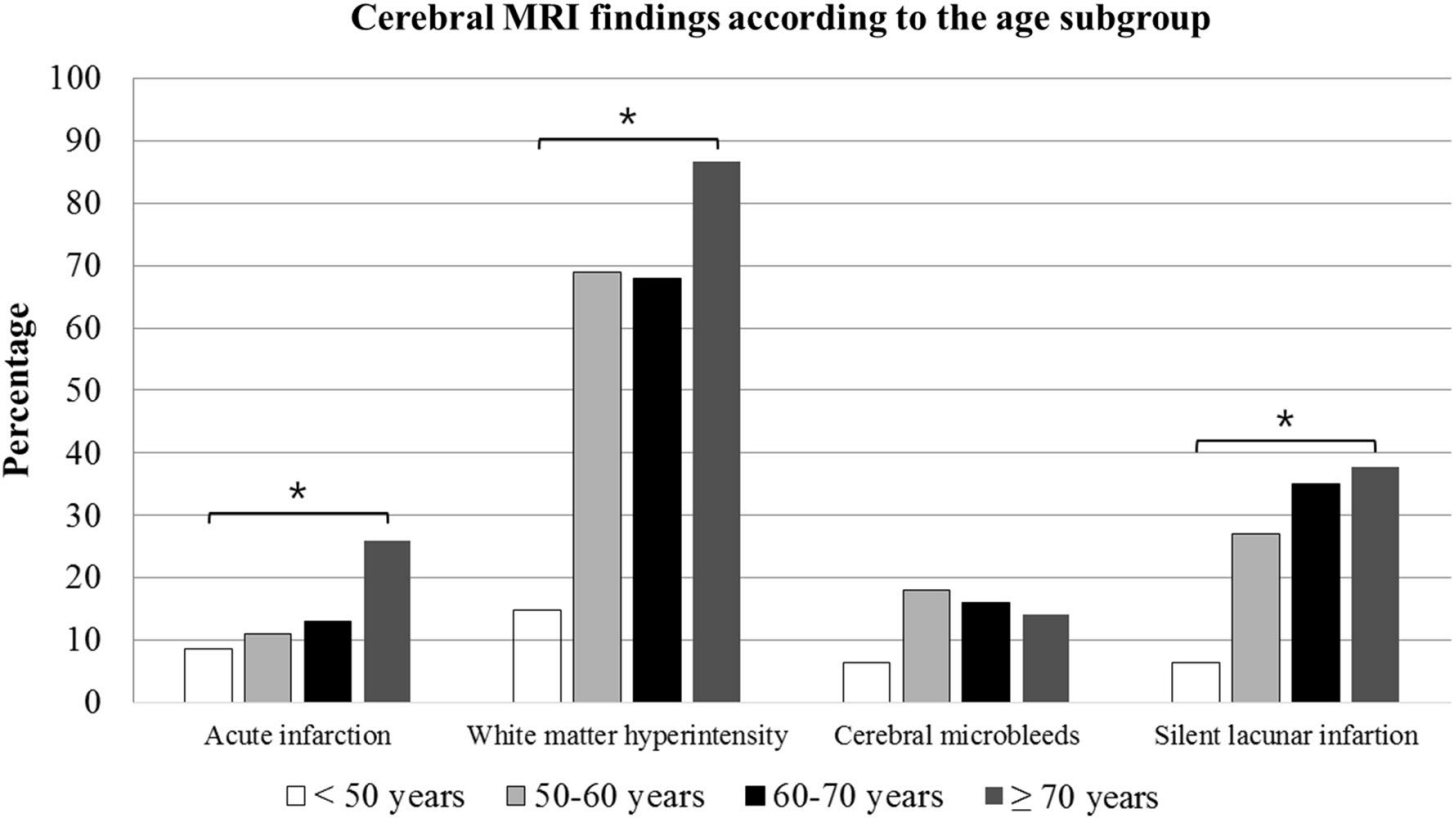
Graphs showing prevalence (%) of cerebral co-incident acute infarction and small vessel disease including white matter hyperintensity lesion, cerebral microbleeds, and silent lacunar infarction by age at the time of cerebral magnetic resonance imaging. The prevalence of cerebral MRI lesions tends to increase with age except cerebral microbleeds. MRI = magnetic resonance imaging. *Significant difference, *P* < 0.05, χ^2^ test.

Table 3 presents the MRI and MRA findings based on age subgroups divided by decades: < 50 years, 50–60 years, 60–70 years, and ≥ 70 years. All categories of the MRI findings demonstrated a significant, increasing trend with age, except for CMB (Fig. 2). MRA also showed the severity of stenosis, which significantly increased with age.

**Table 3:** Comparison of cerebral MRI findings between the age subgroups of < 50 years, 50–60 years, 60–70 years, and ≥ 70 years.

	< 50 years (n = 44)	50–60 years (n = 43)	60–70 years (n = 59)	≥ 70 years (n = 126)	*P* value
Male sex, n (%)	22 (50%)	26 (60%)	47 (80%)	74 (59%)	**0.011**^a^
Comorbidity					
Hypertension, n (%)	8 (18%)	20 (47%)	35 (59%)	100 (79%)	** < 0.001**^**a**^
Diabetes mellitus, n (%)	4 (9%)	7 (16%)	11 (19%)	32 (25%)	0.110^a^
Dyslipidemia, n (%)	10 (23%)	8 (19%)	23 (39%)	38 (30%)	0.110^a^
Obesity, n (%)	12 (27%)	15 (35%)	17 (29%)	27 (21%)	0.334^a^
Smoking, n (%)					
Current smoker	13 (30%)	8 (19%)	9 (15%)	13 (10%)	0.165^b^
Ex-smoker	2 (5%)	8 (19%)	13 (22%)	22 (17%)	
Never	29 (66%)	27 (63%)	37 (63%)	91 (72%)	
Coronary artery disease, n (%)	4 (9%)	3 (7%)	8 (14%)	25 (20%)	0.118^a^
Valvular heart disease or Atrial fibrillation, n (%)	9 (20%)	8 (19%)	7 (12%)	22 (17%)	0.665^a^
History of stroke or TIA, n (%)	1 (2%)	3 (7%)	6 (10%)	17 (13%)	0.165^a^
Brain MRI					
Diffusion weighted image, n (%)	39 (89%)	39 (91%)	48 (81%)	118 (94%)	
Co-incident cerebral infarction, n (%)	3 (8%)	5 (13%)	8 (17%)	33 (30%)	**0.021**^a^
Cerebral small vessel disease, n (%)	11 (25%)	31 (72%)	43 (73%)	113 (90%)	** < 0.001**^a^
White matter hyperintensity, n (%)	7 (16%)	31 (72%)	41 (69%)	109 (87%)	** < 0.001**^a^
Grade 1	7 (16%)	28 (65%)	32 (54%)	61 (48%)	** < 0.001**^b^
Grade 2	0 (0%)	2 (5%)	8 (14%)	39 (31%)	
Grade 3	0 (0%)	1 (2%)	1 (2%)	9 (7%)	
Cerebral microbleeds, n (%)	3 (7%)	8 (19%)	9 (15%)	18 (14%)	0.481
Silent lacunar infarct, n (%)	3 (7%)	11 (26%)	21 (36%)	47 (37%)	**0.002**^a^
MRA—ICA involvement, n (%)	10 (23%)	16 (34%)	28 (47%)	84 (67%)	** < 0.001**^a^
Mild stenosis	4 (9%)	9 (21%)	18 (31%)	46 (37%)	** < 0.001**^b^
Moderate to severe stenosis	6 (14%)	7 (16%)	10 (17%)	38 (30%)	

Data are presented as number (%). *P* < 0.05 was deemed to indicate clinical significance, values in boldface are statistically significant.

MRI = magnetic resonance imaging, MRA = magnetic resonance angiography, ICA = internal carotid artery.

^a^χ^2^ test.

^b^Linear-by-linear association.

We evaluated clinical risk factors associated with co-incident cerebral infarction, WMH, and SLI identified by cerebral MRI (Table 4). In multivariable analysis, older age (≥ 70 years; odds ratio [OR] 6.6; 95% confidence interval [CI], 1.8–25.0; *P* = 0.005) was associated with the presence of cerebral infarction. For the presence of WMH, older age was also significant predictor ([50–60 years; OR, 12.4; 95% CI, 4.3–36.1; *P* < 0.001]; [60–70 years; OR, 10.8; 95% CI, 3.9–30.5; *P* < 0.001]; [≥ 70 years; OR, 31.8; 95% CI, 11.2–90.8; *P* < 0.001]). SLI was significantly associated with older age ([60–70 years; OR, 6.0; 95% CI, 1.6–22.6; *P* = 0.008]; [≥ 70 years; OR, 6.5; 95% CI, 1.8–23.5; *P* = 0.004]). Hypertension was associated with WMH and SLI in univariable analysis, but these associations were not significant in multivariable analysis. Male sex, diabetes, dyslipidemia, and obesity were not significantly associated with cerebral infarction, WMH, and SLI in multivariable analysis.

**Table 4:** Uni- and multi-variable analysis for cerebral infarction, white matter hyperintensity, and silent lacunar infarct based on findings on Cerebral magnetic resonance imaging.

Variables	Cerebral infarction				White matter hyperintensity				Silent lacunar infarct			
	Univariable		Multivariable		Univariable		Multivariable		Univariable		Multivariable	
	OR (95% CI)	*P* value	OR (95% CI)	*P* value	OR (95% CI)	*P* value	OR (95% CI)	*P* value	OR (95% CI)	*P* value	OR (95% CI)	*P* value
Male sex	1.5 (0.8–2.9)	0.249	1.6 (0.8–3.1)	0.211	1.6 (0.9–2.6)	0.095	1.5 (0.8–2.9)	0.201	1.7 (1.0–3.0)	0.056	1.6 (0.9–2.8)	0.129
Hypertension	1.1 (0.6–2.0)	0.838	0.6 (0.3–1.3)	0.242	3.0 (1.8–5.1)	** < 0.001**	1.2 (0.6–2.4)	0.607	2.1 (1.2–3.7)	**0.009**	1.3 (0.7–2.5)	0.432
Diabetes	0.8 (0.3–1.7)	0.495	0.7 (0.3–1.6)	0.355	1.7 (0.9–3.5)	0.127	1.1 (0.5–2.5)	0.808	2.0 (1.1–3.7)	**0.028**	1.6 (0.8–3.1)	0.148
Dyslipidemia	1.0 (0.5–1.9)	0.936	1.0 (0.5–2.1)	0.996	0.9 (0.5–1.5)	0.643	0.6 (0.3–1.3)	0.192	0.9 (0.5–1.7)	0.812	0.8 (0.4–1.4)	0.386
Obesity	0.9 (0.4–1.8)	0.777	1.0 (0.5–2.2)	0.914	1.1 (0.6–2.0)	0.782	1.3 (0.6–2.6)	0.474	1.2 (0.6–2.1)	0.631	1.2 (0.6–2.2)	0.557
**Age**												
< 50 years	–	–	–	–	–	–	–	–	–	–	–	–
50–60 years	1.8 (0.4–8.0)	0.443	2.0 (0.4–9.0)	0.373	13.7 (4.8–39.0)	** < 0.001**	12.4 (4.3–36.1)	** < 0.001**	4.7 (1.2–18.3)	**0.026**	3.9 (1.0–15.6)	0.053
60–70 years	2.1 (5.3–8.6)	0.282	2.4 (0.6–9.9)	0.242	12.0 (4.5–32.1)	** < 0.001**	10.8 (3.9–30.5)	** < 0.001**	7.6 (2.1–27.4)	**0.002**	6.0 (1.6–22.6)	**0.008**
≥ 70 years	4.9 (1.4–16.7)	**0.012**	6.6 (1.8–25.0)	**0.005**	33.9 (13.0–88.2)	** < 0.001**	31.8 (11.2–90.8)	** < 0.001**	8.1 (2.4–27.7)	**0.001**	6.5 (1.8–23.5)	**0.004**

*P* < 0.05 was deemed to indicate clinical significance, values in boldface are statistically significant.

OR = odds ratio, CI = confidence interval.

Table 5 shows a comparison of the demographics, clinical characteristics, and cerebral MRI findings of the patients with NA-RAO according to their etiologic subtypes based on the TOAST criteria. The mean age was significantly different between the three groups; the LAA group was oldest (69.3 ± 11.8 years), followed by the cardioembolism (62.4 ± 16.2 years) and undetermined etiology (60.6 ± 14.6 years) groups. The mean age was significantly higher in the LAA group (69.3 ± 11.8 years) than in the other groups (*P* < 0.001), while there was no significant difference between that of the cardioembolism and undetermined etiology groups. However, there were no significant differences in the clinical features between the etiologic subtypes, such as sex, and conventional cardiovascular risk factors, including hypertension, diabetes mellitus, dyslipidemia, obesity, and smoking history. However, the proportion of patients with a history of ischemic stroke or transient ischemic attack was significantly higher in the LAA group, followed by the cardioembolism and undetermined etiology groups (*P* = 0.028). Cerebral infarction was significantly more prevalent in the LAA (n = 33/114; 30%) group, followed by the cardioembolism (n = 7/36; 19%) and undetermined etiology (n = 9/93; 8%) groups (*P* = 0.003). The overall cerebral SVD was also significantly more prevalent in the LAA (n = 103/126; 82%) group, followed by the cardioembolism (n = 26/37; 70%) and undetermined etiology (n = 69/107; 64%) groups (*P* = 0.011). The WMH was more common in the LAA (77%) group, followed by undetermined etiology (64%) and cardioembolism (62%) groups. However, the grade 3 WMH was more common in the cardioembolism (38%) than in the LAA (22%) and undetermined etiology (5%) groups. SLI was also more common in the LAA (41%) group, followed by the cardioembolism (24%) and undetermined etiology (20%) groups.

**Table 5:** Comparison of clinical characteristics and cerebral MRI findings by etiologic subtype between large artery atherosclerosis, cardioembolism, and undetermined in patients with retinal artery occlusion.

	Large artery atherosclerosis (n = 126)	Cardioembolism (n = 37)	Undetermined (n = 107)	*P* value
Age (year), mean ± SD	69.3 ± 11.8	62.4 ± 16.2^c^	60.6 ± 14.6^c^	** < 0.001**^a^
Male sex, n (%)	83 (66%)	23 (62%)	63 (59%)	0.545
Time from symptom onset to initial brain imaging (day) , mean ± SD	4.1 ± 7.5	3.3 ± 6.7	4.8 ± 7.9	0.572
Comorbidity				
Hypertension, n (%)	85 (67%)	18 (49%)	60 (56%)	0.061
Diabetes mellitus, n (%)	33 (26%)	5 (14%)	16 (15%)	0.058
Dyslipidemia, n (%)	42 (33%)	7 (19%)	30 (28%)	0.223
Obesity, n (%)	28 (22%)	11 (30%)	31 (29%)	0.428
Smoking, n (%)				
Current smoker	24 (19%)	2 (5%)	17 (16%)	0.409^b^
Ex-smoker	20 (16%)	7 (19%)	18 (17%)	
Never	82 (65%)	28 (76%)	72 (67%)	
Coronary artery disease, n (%)	18 (14%)	10 (27%)	12 (11%)	0.064
History of ischemic stroke or TIA	19 (15%)	3 (8%)	5 (5%)	**0.028**
Brain MRI				
Diffusion weighted image, n (%)	114 (90%)	36 (97%)	93 (87%)	
Co-incident cerebral infarction, n (%)	33 (30%)	7 (19%)	9 (8%)	**0.003**
Cerebral small vessel disease	103 (82%)	26 (70%)	69 (64%)	**0.011**
White matter hyperintensity, n (%)	97 (77%)	23 (62%)	68 (64%)	**0.048**
Grade 1	32 (25%)	4 (11%)	50 (47%)	**0.015**^b^
Grade 2	60 (48%)	18 (49%)	13 (12%)	
Grade 3	28 (22%)	14 (38%)	5 (5%)	
Cerebral microbleeds, n (%)	15 (12%)	8 (22%)	15 (14%)	0.327
Silent lacunar infarct, n (%)	52 (41%)	9 (24%)	21 (20%)	**0.001**

Data are presented as number (%) or mean ± standard deviation. *P* < 0.05 was deemed to indicate clinical significance, values in boldface are statistically significant.

MRI = magnetic resonance imaging, TIA = transient ischemic attack.

^a^Analysis of variance.

^b^Linear-by-linear association.

^c^Nonsignificant difference (*P* > 0.05).

## Discussion

This study investigated cerebral MRI findings at the time of diagnosis in a large group of patients with NA-RAO. Co-incident cerebral infarctions and cerebral SVD were observed in 18% and 73% of patients with RAO, respectively. WMH (70%) was the most common type of SVD, followed by SLI (30%) and CMB (14%). The embolic etiology of NA-RAO was significantly associated with the prevalence of co-incident infarction and SVD, and the prevalence was higher in LAA etiology than cardioembolism and undetermined etiology. Older age, in particular 70 years or older, was an independent predictor for the presence of co-incident cerebral infarction, WMH, and SLI. Considering the disease burden and need for proper management of ischemic stroke and cognitive disorders^6,20,39^, it is imperative to conduct an appropriate systemic evaluation, which includes cerebral MRI/MRA with DWI, particularly in elderly patients with NA-RAO.

NA-RAO may require appropriate evaluation and management similar to a stroke^6^. NA-RAO and ischemic strokes are closely related in terms of temporal and etiological aspects^2,8,40,41^. Retinal and ophthalmic arteries branch from the ICA, the latter which also supplies blood to the brain^7^. When thromboembolism occurs in the large artery or the heart, it can enter the eye or brain through the ICA. Therefore, thromboembolic events can occur via the same mechanism in the retina and brain^6,7^. Studies have reported an increased risk of subsequent stroke after RAO, particularly within a month^8,11,42,43^. Recently, there has been controversy as to whether emergency neurologic evaluation is necessary in patients with RAO^12,13,44^. Most experts agree that the evaluation of embolic sources in patients with RAO is essential. However, it is unclear whether neurological evaluation, including MRI, should be performed in an emergent situation. Hayreh suggested that it is not necessary to have neurological counseling at the stroke center or emergency room in the absence of additional neurological findings^12^. Alternatively, Lavin et al*.* suggested that evaluation of stroke should be performed immediately due to its high incidence (18%), particularly during and after RAO. In addition, one meta-analysis reported that 30% of patients with acute CRAO and 25% of patients with acute BRAO presented an acute cerebral ischemia on MRI^10^. Based on our results and those of previous studies, urgent brain imaging, including DWI, should be considered in patients with NA-RAO. MRI is often performed in referral hospitals and is relatively expensive. Since the accessibility and coverage of healthcare systems vary across countries, MRI sometimes may not be performed immediately. However, considering the prevalence (27–76.4% of patients with CRAO) of co-incident stroke in patients with NA-RAO^2,6,9,11^, we believe that urgent MRI/MRA with DWI is needed to reduce morbidity and mortality. In particular, since the incidence of SVD and concomitant brain diseases is high in elderly patients^19^, it is necessary to evaluate and manage these patients. Further studies are needed to assess the cost–benefit of cerebral MRI.

Although there have been several reports that have suggested that RAO may increase the incidence of subsequent stroke, definite evidence that neurological examination, including MRI, would facilitate the management of patients with RAO is lacking. Previous SVD studies show that WMH, SLI, and CMB are related to future stroke and mortality^24,26,45^; therefore, SVD found in patients with NA-RAO may be helpful for prognosis prediction. It has been suggested that injury and inflammation of cerebral vessels and cells (smooth muscle cells or oligodendrocytes, etc.) in SVD may result in vessel fragility and endothelial instability, with hemorrhagic or ischemic consequences^23^. In a meta-analysis of six population-based studies, there was a significant association of WMH with risk of stroke (hazard ratio: 3.1, 95% CI: 2.3–4.1, *P* < 0.001)^20^. In a study involving 1096 Korean patients with acute ischemic stroke, 26.8% had CMB, 16.4% had high-grade WMH (Grade 3 or ≥ 2 in deep white matter), and 38% had SLI^45^. In another cohort study of 500 patients with transient ischemic attack in the Korean Transient Ischemic Attack Expression Registry showed 30% acute DWI lesions, 31.8% WMH, and 8.6% CMB, which was comparable to our study^46^. In a previous study that evaluated the prevalence of cerebral SVD in the ischemic stroke population (median age, 68.1; male sex, 62%), the prevalence of WMH, SLI, and CMB was 37%, 20%, and 15%, respectively^47^. Although there were differences in demographics between the populations with ischemic stroke and NA-RAO, which made it difficult to make a clear comparison, the NA-RAO group showed a similar, or a relatively greater, prevalence of SVD than the stroke patient group. In addition, patients with WMH and CMB had a significantly higher incidence of stroke within 90 days^46^. Furthermore, despite the meticulous examination, the embolic source was not confirmed in 39% of patients with NA-RAO in our study. The need for medical intervention in the undetermined etiology group may be overlooked; however, most of these patients demonstrated the presence of cerebral SVD, although the SVD ratio in the undetermined etiology group was lower than that of LAA or cardioembolism. Since SVD increases the risk of ischemic stroke^20^, more active examination and management may be considered if a patient with an undetermined embolic origin has SVD.

In addition to ischemic stroke, a comprehensive approach to cognitive disorders associated with SVD may help provide general care to patients^21,48^. SVD is associated with cognitive decline and, ultimately, dementia^20,39^ (up to 45% of all dementias^49^), and a meta-analysis showed that both WMH and SLI are associated with an increased risk of dementia (hazard ratio: 1.48, 95% CI: 1.10–1.99 and hazard ratio: 1.56, 95% CI: 1.10–2.23, respectively)^27^. SVD could also cause parkinsonism and mood disorders^50,51^. However, these gradual declines in cognitive, emotional, and motor functions due to SVD are often ignored, since they do not cause sudden disability like ischemic stroke. Since SVD has a significant effect on health in the elderly, much attention is being paid to therapeutic management. Several drugs, such as antiplatelet agents, anti-inflammatory agents, and anti-dementia drugs are being used for SVD; however, there are currently no established therapeutic strategies^52^. Vascular prophylaxis, as appropriate for large artery and cardiac thromboembolism, includes antithrombotics, and blood pressure and lipid lowering agents are typically used in primary care treatment of SVD^52^.

The main etiology of NA-RAO is thromboembolism that originates from the LAA and heart^2,53^. Previous studies showed that the direction of the embolus depends on its origin, size, and density^6,53,54^. In our study, the prevalence of cardioembolism was significantly higher in the CRAO than in the BRAO, possibly due to the relatively large size of the cardiac emboli^53^. However, emboli derived from LAA were not significantly different between the CRAO and BRAO, suggesting that embolism of various sizes may have randomly entered the ophthalmic artery. In this study, there was no difference in cerebral MRI findings based on RAO type, since the CRAO and the BRAO may have common pathological mechanisms. In contrast, coincident cerebral infarcts, WMH, and SLI were significantly more common in the LAA group than in the cardioembolism and undetermined etiology groups. Since there were differences in age and underlying diseases, comparisons by etiology group were unavailable in our study. Interestingly, the mean age of the cardioembolism group (62.4 ± 16.2 years; range 20–85 years) was lower than that of LAA group (69.3 ± 11.8 years, range 32–93 years) (*P* < 0.001), suggesting that a thorough cardiac examination is necessary in the future, especially for young patients. Considering the fact that LAA was associated with the presence of a subsequent vascular event in previous studies^2^, it may be associated with cerebral MRI findings. However, this requires further investigation.

The incidence and prevalence of stroke and SVD increased with age. The incidence of stroke doubled for each decade after an age of 55^55^. The prevalence of WMH and CMB increased significantly with age in healthy subjects without major cerebrovascular risk factors^56^. In the Framingham Heart Study, the prevalence of infarction and WMH volume observed in cerebral MRI was significantly associated with age^57^. In an Asian population-based study from Singapore, Hong Kong, and Korea, the presence of three SVD markers (WMH, CMB, and SLI) showed a significant increase with increasing age, rising from 1.9% in ages 60–64 years to 46.2% in those aged 75 years and above^58^. Therefore, there was a significant positive correlation of the prevalence of co-incident acute infarction, WMH, and SLI with the age in patients with NA-RAO in our study, as in previous studies. Considering that co-incident infarction and SVD increase with age, neurologic examination and cerebral MRI are important assessments, particularly in elderly patients with NA-RAO.

Our study has several limitations. First, the time from the onset of symptoms to the first ophthalmologic diagnosis varied from 1–31 days, which may have affected the cerebral MRI findings, especially co-incident cerebral infarction. There was also a difference in the timing of MRI of BRAO and CRAO, which may have influenced MRI findings between the two groups of patients. However, a significant cerebral infarction usually resulted in neurological symptoms, highlighting the need for a cerebral MRI scan, which could confirm the presence of an acute stroke. In addition, most patients (83%) underwent cerebral MRI within 7 days of symptom onset. DWI demonstrated high signal intensity 10–14 days after ischemic stroke, after which it showed variable signal intensity in the chronic phase (> 3 weeks)^59^. Therefore, in the subacute phase within 10 days, most acute strokes can be detected through DWI. Second, due to the nature of the cross-sectional study, it is not known whether SVD is an additional risk factor for systemic vascular events or mortality in patients with NA-RAO, and further longitudinal studies are needed. Third, our patient population was Korean; therefore, our study findings need to be validated among other ethnicities before generalization. Fourth, this is a retrospective study, which inherently has a potential selection bias. Despite these limitations, our study showed that a large number of patients with RAO could be enrolled, and the detailed cerebral MRI findings were evaluated and analyzed; therefore, our results were robust.

Our cerebral MRI findings in patients with NA-RAO not only detected co-incident cerebral infarction, but also a large number of cerebral SVD findings, including WMH, SLI, and CMB. Furthermore, the types of RAO (central vs. branch) were not associated with MRI findings. Co-incident acute cerebral infarction, WMH, and SLI significantly increased with age. There was significantly more coincident cerebral infarction, WMH, and SLI in the LAA group than in the cardioembolism and undetermined etiology groups. The high co-incidence of acute cerebral infarction and underlying SVD burden warrant careful neurologic examination and appropriate brain imaging with management of NA-RAO, especially in elderly patients.

## Supplementary Information

Supplementary Information.

## Data Availability

The datasets generated and/or analyzed during the current study are available from the corresponding author upon reasonable request.

## Abbreviations

NA-RAO: Non-arteritic retinal artery occlusion
CRAO: Central RAO
BRAO: Branch RAO
MRI: Magnetic resonance imaging
SVD: Small vessel disease
WMH: White matter hyperintensity
CMB: Cerebral microbleed
SLI: Silent lacunar infarction
DWI: Diffusion weighted image
SNUBH: Seoul National University Bundang Hospital
