# Supplementary material for: Cerebral magnetic resonance imaging of coincidental infarction and small vessel disease in retinal artery occlusion
Source: Sci Rep. 2021 Jan 13;11:864. doi: 10.1038/s41598-020-80014-9 (PMC7806736; doi:10.1038/s41598-020-80014-9)
Supplement: Supplementary file 1 — Supplementary Information. [file 41598_2020_80014_MOESM1_ESM.pdf]

Cerebral magnetic resonance imaging of coincidental infarction and small vessel disease in retinal artery occlusion

Yong Dae Kim[1, 2]*, Jun Yup Kim[3, 4]*, Young Joo Park[5], Sang Jun Park[1], Sung Hyun Baik[3], Jihoon Kang[4], Cheolkyu Jung[3], Se Joon Woo[1]

[1]Department of Ophthalmology, Seoul National University College of Medicine, Seoul National University Bundang Hospital, Seongnam, Korea

[2]Department of Ophthalmology, Kangdong Sacred Heart Hospital, Seoul, Korea

[3]Department of Radiology, Seoul National University College of Medicine, Seoul National University Bundang Hospital, Seongnam, Korea

[4]Department of Neurology, Seoul National University College of Medicine, Seoul National University Bundang Hospital, Seongnam, Korea

[5]Department of Ophthalmology, Kangwon National University Hospital, Chuncheon, South Korea

*These authors contributed equally to this work.

Supplementary Table S1. Comparison of clinical characteristics and brain MRI findings between patients with complete central retinal artery occlusion (CRAO) and patients with incomplete CRAO.

Variables	Complete CRAO (n = 151)	Incomplete CRAO (n = 39)	*P* value
Age (year), mean ± SD	62.6 ± 16.2	65.6 ± 13.4	0.230
Male sex, n (%)	89 (59%)	25 (64%)	0.557
Time from symptom onset to initial brain imaging (day) , mean ± SD	2.1 ± 2.7	3.4 ± 6.6	0.216
Comorbidity			
Hypertension, n (%)	91 (60%)	18 (46%)	0.112
Diabetes mellitus, n (%)	36 (24%)	5 (13%)	0.136
Dyslipidemia, n (%)	45 (30%)	11 (28%)	0.845
Obesity, n (%)	38 (25%)	8 (21%)	0.545
Smoking, n (%)			
Current smoker	99 (66%)	27 (69%)	0.690[a]
Ex-smoker	26 (17%)	6 (15%)	
Never	26 (17%)	6 (15%)	
Coronary artery disease, n (%)	29 (19%)	6 (15%)	0.583
Embolic heart disease or Atrial fibrillation, n (%)	26 (17%)	6 (15%)	0.785
History of ischemic stroke or TIA	14 (9%)	7 (18%)	0.123
Brain MRI/MRA			
Diffusion weighted image, n (%)	137 (91%)	37 (95%)	
Co-incident cerebral infarction, n (%)	30 (22%)	7 (19%)	0.694
Cerebral small vessel disease	115 (76%)	27 (69%)	0.375
White matter hyperintensity, n (%)	112 (74%)	24 (62%)	0.119
Grade 1	80 (53%)	16 (41%)	0.189[a]
Grade 2	24 (16%)	8 (21%)	
Grade 3	8 (5%)	0 (0%)	
Cerebral microbleeds, n (%)	22 (15%)	6 (15%)	0.801
Silent lacunar infarct, n (%)	46 (30%)	13 (33%)	0.730
MRA - ICA involvement			
Mild stenosis	39 (26%)	13 (33%)	0.274
Moderate to severe stenosis	37 (25%)	7 (18%)	

Data are presented as number (%) or mean ± standard deviation. $P < 0.05$ was deemed to indicate clinical significance, values in boldface are statistically significant.

MRI = magnetic resonance imaging, TIA = transient ischemic attack, MRA = magnetic resonance angiography, ICA = internal carotid artery.

[a]Linear-by-linear association
